# Glycopyrrolate/formoterol fumarate metered dose inhaler for maintenance-naïve patients with chronic obstructive pulmonary disease: a *post-hoc* analysis of the randomized PINNACLE trials

**DOI:** 10.1186/s12931-020-1332-3

**Published:** 2020-03-12

**Authors:** Jinping Zheng, Jin-fu Xu, Martin Jenkins, Pryseley Nkouibert Assam, Lijiao Wang, Brian J. Lipworth

**Affiliations:** 1 State Key Laboratory of Respiratory Disease, National Clinical Research Center of Respiratory Disease, Guangzhou Institute of Respiratory Health, First Affiliated Hospital of Guangzhou Medical University, Guangzhou, China; 2 Department of Respiratory and Critical Care Medicine, Shanghai Pulmonary Hospital, Tongji University School of Medicine, Shanghai, China; 3 Global Medicines Development, AstraZeneca, Central Cambridge, UK; 4 Global Medicines Development, AstraZeneca, Shanghai, China; 5 Medical Affairs, AstraZeneca, Shanghai, China; 6 Scottish Centre for Respiratory Research, Ninewells Hospital, University of Dundee, Dundee, DD1 9SY Scotland, UK

**Keywords:** Glycopyrrolate/formoterol fumarate, Chronic obstructive pulmonary disease, Long-acting bronchodilator-naïve

## Abstract

**Background:**

Glycopyrrolate (GP)/formoterol fumarate (FF; GFF) metered dose inhaler is a fixed-dose combination dual bronchodilator for patients with chronic obstructive pulmonary disease (COPD); however, whether the efficacy in patients without current maintenance treatment is consistent with currently maintenance-treated patients is unclear.

**Methods:**

Data from patients who were not maintenance-treated at screening (NMT) (*n* = 1943) and patients who were maintenance-treated at screening (MT) patients (*n* = 3040) receiving GFF, FF, GP, or placebo were pooled from the Phase III PINNACLE studies (NCT01854645, NCT01854658, NCT02343458) for *post-hoc* analysis. MT patients had received long-acting bronchodilators and/or inhaled corticosteroids in the 30 days prior to screening, and/or prior to randomization. NMT patients had received short-acting bronchodilators or no treatment. Outcomes included forced expiratory volume over 1 s (FEV_1_), clinically important deterioration (CID), rescue medication use, and safety.

**Results:**

GFF provided significant lung function improvements at Week 24 versus placebo, GP, and FF for NMT patients, with pre-dose trough FEV_1_ treatment differences of 152 (117–188) mL, 73 (45–100) mL, and 56 (29–84) mL, respectively (least squares mean change from baseline versus comparators [95% CI]; all *P* < 0.0001). GFF reduced the risk of CID by 17–43% in NMT (*P* ≤ 0.0157) and 18–52% (*P* ≤ 0.0012) in MT patients compared with monotherapy and placebo, and reduced rescue medication use by 1.5 puffs/day over 24 weeks for both cohorts. Safety profiles for all cohorts were consistent with each other and the parent studies.

**Conclusions:**

NMT patients achieved better lung function with GFF versus monotherapy and placebo, without increased safety risk. Dual bronchodilator therapy may offer better outcomes than monotherapy for COPD patients when administered as first-line treatment.

## Background

Long-acting bronchodilators are recommended as first-line maintenance therapy for chronic obstructive pulmonary disease (COPD) to control symptoms and prevent exacerbations [1]. Currently, monotherapy with either a long-acting muscarinic antagonist (LAMA) or long-acting beta-agonist (LABA) is preferred, the exception being for stage B patients as per the Global Initiative for Chronic Obstructive Lung Disease (GOLD B patients) who experience severe breathlessness, as well as highly symptomatic GOLD D patients. For these groups, LAMA/LABA or LABA/inhaled corticosteroid (ICS) combinations are indicated [1]. In patients with persistent dyspnea, exacerbations, or exercise limitations despite monotherapy, step-up treatment to a LAMA/LABA combination is advised [1].

Limited evidence is available to guide the choice of first-line maintenance therapy in COPD [1]. Studies of maintenance treatment-naïve patients treated with monotherapy have found safety and efficacy outcomes to be consistent with unsorted populations, confirming that they are an appropriate first-line option [2–4]. However, many patients treated with monotherapy continue to experience COPD symptoms, exacerbations, and poor quality of life [5, 6]. In randomized trials, LAMA/LABA combinations consistently provide superior lung function and exercise capacity improvements, and better symptom reduction than respective monocomponents [7–9]. A systematic review of LABA/LAMA versus LABA, LAMA, and LABA/ICS examined 27 studies to find significantly improved respiratory outcomes with dual LAMA/LABA therapy [9]. Commencing new patients directly on LAMA/LABA combinations may improve outcomes, irrespective of GOLD stage [10–12]. However, there is limited evidence clarifying whether dual therapy is appropriate for the initial treatment of COPD as few studies have been conducted in patients with lapsed or no prior history of maintenance therapy.

Glycopyrrolate/formoterol fumarate (GFF) metered dose inhaler (MDI) is a fixed-dose combination LAMA/LABA bronchodilator administered in a single Aerosphere inhaler using innovative co-suspension delivery technology [13, 14]. In the PINNACLE trials, GFF MDI achieved superior respiratory outcomes compared with monotherapy and placebo in patients with COPD across diverse treatment backgrounds [13, 14]. GFF MDI is currently approved for long-term COPD maintenance therapy in the USA, Europe, Canada, Australia, Japan, and South Korea, among others.

To assess whether GFF MDI is appropriate for initial maintenance treatment in COPD, we conducted a *post-hoc* analysis comparing GFF to LAMA and LABA monocomponents and placebo in patients not receiving maintenance treatment at screening (NMT) and patients receiving maintenance treatment at screening (MT) pooled from three randomized phase III trials.

## Methods

### Study design

This study was a pooled, *post-hoc* analysis of three randomized, multicenter, international, double-blind, 24-week Phase III clinical trials, PINNACLE-1 (NCT01854645), -2 (NCT01854658), and -4 (NCT02343458), which compared the efficacy of GFF MDI with its monocomponents and placebo. The study details and primary outcomes have previously been published [13, 14]. Briefly, patients were randomized to receive twice-daily GFF MDI 14.4/9.6 μg, glycopyrrolate (GP) MDI 14.4 μg, formoterol fumarate (FF) MDI 9.6 μg, or placebo MDI. PINNACLE-1 included a tiotropium arm, which has been excluded from our analysis. Patients provided signed informed consent prior to screening. The studies were conducted in accordance with the amended Declaration of Helsinki and approved by local institutional review boards (details previously published) [13, 14].

### Participants

Enrolled subjects were aged > 40 years, from the USA, Australia, New Zealand, Europe, and Asia (including China and Japan), and current or former smokers (≥10 packs/year). They also had clinical history of moderate-to-very severe COPD, defined per the American Thoracic Society/European Society criteria as patients with post-bronchodilator forced expiratory volume over 1 s (FEV_1_)/forced vital capacity ratio < 0.70, and FEV_1_ < 80% predicted. Subjects were retrospectively classified by treatment history. MT patients were those who received any maintenance therapy (ICS, LAMA, LABA, or combinations) during the 30 days prior to screening or prior to randomization. NMT patients had received short-acting bronchodilators or remained untreated.

### Outcomes and procedures

Lung function was assessed by change from baseline in morning pre-dose trough FEV_1_ at Week 24, a primary endpoint of all three parent studies. Peak change from baseline in FEV_1_ within 2 h post-dosing at Week 24 was a secondary endpoint. Spirometry was performed in accordance with the American Thoracic Society criteria. Other secondary endpoints included time to first clinically important deterioration (CID), health-related quality of life as assessed by St George’s Respiratory Questionnaire (SGRQ), and symptom burden as assessed by rescue salbutamol use over 24 weeks. First CID was defined as the first occurrence of either ≥100 mL decline in trough FEV_1_, treatment-emergent moderate or severe COPD exacerbation, or increase of ≥4.0 units on the SGRQ. Safety was assessed by adverse event monitoring. Subgroup analyses were performed for the NMT cohort in patients who were Chinese and patients who were symptomatic (baseline COPD Assessment Test [CAT] score ≥ 15).

### Statistical analysis

Separate efficacy analyses were conducted within the NMT and MT groups. Patients were drawn from the intent-to-treat (ITT) populations of the parent trials, defined as subjects who were randomized and received ≥1 dose of treatment (excluding tiotropium). Safety assessments utilised the safety population of the parent trials. Change from baseline in trough FEV_1_ was analyzed using a repeated measures linear model that included baseline and reversibility to salbutamol as continuous covariates, and study, treatment, visit, and treatment by visit interaction as categorical covariates. Similar repeated measures models were used for analyzing peak FEV_1_, SGRQ, and salbutamol use. Time to first CID was analyzed using a Cox regression model adjusted for baseline percentage-predicted FEV_1_, baseline CAT score, baseline eosinophil count, study, exacerbation history in the previous year, smoking status, and baseline ICS use. For all analyses, pairwise treatment effect estimates were produced with 95% confidence intervals (CIs) and *P*-values. These analyses were defined *post-hoc*, thus no adjustment for multiplicity was made, and they were not prospectively powered. *P*-values should be regarded as exploratory, and interpreted in terms of nominal significance.

## Results

The pooled ITT population consisted of 4983 patients (GFF MDI, *n* = 1585; FF MDI, *n* = 1360; GP MDI, *n* = 1362; placebo MDI, *n* = 676), all of whom were included for efficacy analyses. Patients were classified as NMT (*n* = 1943) or MT (*n* = 3040) (Table 1). Of the NMT patients 10.0% were Chinese and 64.7% were symptomatic. Similar demographics and baseline characteristics were broadly observed between the NMT and MT cohorts, and all treatment subgroups (Table 1; individual treatment groups not shown). There was a higher proportion of current smokers and subjects with moderate-severity COPD in the NMT cohort, compared to the MT cohort. Not unexpectedly, MT patients had longer treatment duration, and were more likely to be GOLD D stage than NMT patients.

**Table 1:** Demographics and baseline characteristics

	NMT (***n*** = 1943)	MT (***n*** = 3040)
**Age, mean (SD), years**	61.3 (8.2)	64.6 (7.8)
**Sex, n (%)**		
Male	1193 (61.4)	1893 (62.3)
**Race, n (%)**		
White	1563 (80.4)	2381 (78.3)
Asian	229 (11.8)	482 (15.9)
Black or African American	139 (7.2)	163 (5.4)
Other	12 (0.6)	14 (0.4)
**Smoking status**		
Current, n (%)	1244 (64.0)	1284 (42.2)
Former, n (%)	699 (36.0)	1756 (57.8)
Number of packs/year smoked, mean (SD)	49.5 (25.9)	49.1 (26.2)
**CAT score**		
Total CAT score, mean (SD)	17.8 (7.7)	17.0 (7.4)
Symptomatic (CAT ≥15), n (%)	1257 (64.7)	1825 (60.0)
**mMRC grade, mean (SD)**	1.7 (1.0)	1.8 (0.9)
**COPD severity, n (%)**		
Mild (GOLD 1)	13 (0.7)	17 (0.6)
Moderate (GOLD 2)	1230 (63.3)	1534 (50.5)
Severe (GOLD 3)	644 (33.1)	1327 (43.7)
Very severe (GOLD 4)	56 (2.9)	162 (5.3)
**GOLD 2017 category, n (%)**		
A	276 (14.2)	453 (14.9)
B	1516 (78.0)	2177 (71.6)
C	19 (1.0)	54 (1.8)
D	125 (6.4)	348 (11.4)
Missing	7 (0.4)	8 (0.3)
**COPD duration, mean (SD), years**	6.5 (6.3)	7.6 (6.2)

*CAT* COPD Assessment Test, *COPD* Chronic obstructive pulmonary disease, *GOLD* Global Initiative for Chronic Obstructive Lung Disease, *mMRC* Modified Medical Research Council scale, *SD* Standard deviation

**Fig. 1:**
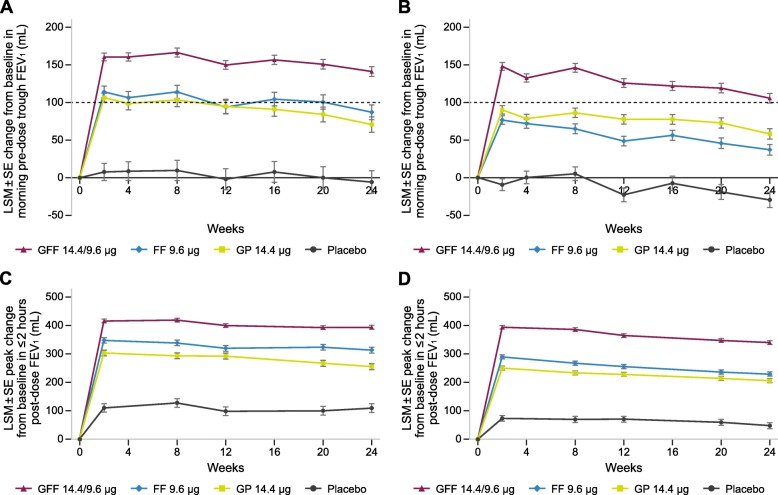
Pre-dose trough and peak post-dose FEV_1_ for NMT (**a**, **c**) and MT (**b**, **d**) patients. *P* < 0.0001 for all comparisons excluding FF versus GP. All *P*-values < 0.05 should only be interpreted in terms of nominal significance as the analyses in these subgroups were exploratory and post-hoc. Interrupted line at 100 mL indicates MCID for change in trough FEV_1_ (1A and 1B). COPD = chronic obstructive pulmonary disease; FEV_1_ = forced expiratory volume over 1 s; FF = formoterol fumarate; GFF = glycopyrrolate/formoterol fumarate; GP = glycopyrrolate; LSM = least squares mean; MCID = minimal clinically important difference; MT = maintenance-treated at screening; NMT = not maintenance-treated at screening; SE = standard error

Treatment with GFF MDI provided significant lung function improvements at Week 24 versus placebo, FF, and GP for NMT patients, with change from baseline treatment differences in morning pre-dose trough FEV_1_ of 152 mL, 56 mL, and 73 mL, respectively (all *P* < 0.0001) (Fig. 1a). The MT cohort had similar improvements with GFF versus placebo, FF, and GP (treatment differences of 140 mL, 71 mL, and 49 mL, respectively; all *P* < 0.0001) (Fig. 1b). Significant differences between GFF and all comparators were observed as early as Week 2 and maintained over 24 weeks. Similar improvements were observed with GFF versus placebo, FF, and GP for peak change from baseline in FEV_1_ within 2 h post-dose at Week 24 (treatment differences for NMT: 283 mL, 79 mL, and 138 mL, respectively; and MT: 291 mL, 109 mL, and 133 mL, respectively; all *P* < 0.0001) (Fig. 1c–d). These findings were consistent with the overall ITT population.

**Fig. 2:**
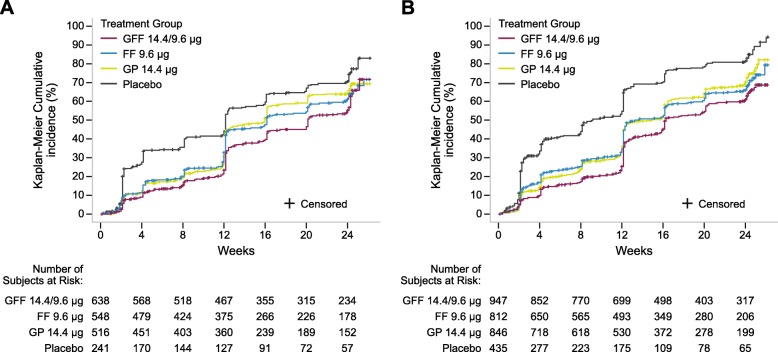
Time to first CID event for NMT (**a**) and MT (**b**) patients. Time to CID (weeks) = (date of CID – first treatment administration date + 1)/7. CID was defined as the first occurrence of one of the following events: ≥100 mL decline in trough FEV_1,_ treatment-emergent moderate or severe COPD exacerbation, or increase of ≥ 4.0 units in SGRQ score. CID = clinically important deterioration; COPD = chronic obstructive pulmonary disease; FEV_1_ = forced expiratory volume in 1 s; FF = formoterol fumarate; GFF = glycopyrrolate/formoterol fumarate; GP = glycopyrrolate; MDI = metered dose inhaler; MT = maintenance-treated at screening; NMT = not maintenance-treated at screening; SGRQ = St George’s Respiratory Questionnaire

GFF MDI improved CID measures for all patients. For NMT patients, GFF MDI reduced the risk of CID by 43, 21, and 17% versus placebo, GP, and FF, respectively (Table 2). In the MT cohort, CID risk reduction with GFF MDI was 52, 23, and 18%, respectively, as above. Median time to first CID, measured in weeks, was longer for GFF (20.1) versus placebo (12.1), GP (16.0), and FF (16.1) for NMT patients (Fig. 2; Table 2). Increased time to first CID with GFF MDI was similarly observed in the MT cohort. Not unexpectedly, due to the higher proportion of GOLD D patients and with increased COPD severity, median time to CID was shorter in the MT cohort compared to NMT patients across all treatment groups.

**Table 2:** Clinically important deterioration (CID) and patient-reported outcomes

	NMT				MT			
Treatment	GFF	FF	GP	Placebo	GFF	FF	GP	Placebo
**Time to first CID**								
Patients with CID, n/N (%)	349/638 (54.7)	331/548 (60.4)	323/516 (62.6)	169/241 (70.1)	576/947 (60.8)	514/812 (63.3)	576/846 (68.1)	334/435 (76.8)
Median time to CID, weeks	20.1	16.1	16.0	12.1	16.3	13.3	14.9	9.1
Hazards ratio vs GFF MDI (95% CI)	–	0.83 (0.71, 0.97)	0.79 (0.67, 0.91)	0.57 (0.48, 0.69)	–	0.82 (0.73, 0.92)	0.77 (0.68, 0.86)	0.48 (0.42, 0.55)
*P*-value	–	0.0157	0.0018	< 0.0001	–	0.0012	< 0.0001	< 0.0001
**SGRQ total score at Week 24**								
n	528	465	436	200	814	668	681	319
Baseline score, mean (SD)	45.00 (17.52)	45.63 (18.30)	44.29 (18.65)	45.34 (17.83)	45.25 (17.54)	43.24 (17.52)	44.51 (17.95)	44.79 (17.94)
Change from baseline, LSM (SE)	−4.40 (0.52)	−5.21 (0.56)	−4.01 (0.58)	−2.65 (0.86)	−4.13 (0.41)	−3.29 (0.46)	−1.954 (0.45)	−0.86 (0.66)
Treatment difference vs GFF MDI (95% CI)	–	0.81 (− 0.69, 2.31)	− 0.39 (− 1.92, 1.14)	−1.75 (− 3.71, 0.22)	–	− 0.83 (− 2.04, 0.37)	−2.17 (− 3.36, − 0.99)	−3.26 (− 4.78, − 1.75)
*P*-value	–	0.2903	0.6179	0.0810	–	0.1735	0.0003	< 0.0001
**Rescue salbutamol use over 24 weeks (rescue medication user analysis set)**								
n	347	312	299	138	565	492	519	276
Change from baseline in rescue salbutamol use (SE), puffs/day	−1.5 (0.13)	−1.4 (0.14)	− 1.3 (0.14)	−0.6 (0.21)	−1.5 (0.10)	− 1.1 (0.11)	− 0.6 (0.11)	0.01 (0.15)
Treatment difference vs GFF MDI (95% CI), puffs/day	–	−0.2 (− 0.5, 0.2)	−0.2 (− 0.6, 0.2)	−0.9 (− 1.4, − 0.5)	–	−0.4 (− 0.7, − 0.1)	−0.8 (− 1.1, − 0.6)	− 1.5 (− 1.9, − 1.2)
*P*-value	–	0.3845	0.2740	< 0.0001	–	0.0046	< 0.0001	< 0.0001

All *P*-values < 0.05 should only be interpreted in terms of nominal significance as the analyses in these subgroups were exploratory and post-hoc. Numbers rounded to 2 decimal places. *CI* Confidence interval, *FF* Formoterol fumarate, *GFF* Glycopyrrolate/formoterol fumarate, *GP* Glycopyrrolate, *LSM* Least squares mean, *MDI* Metered dose inhaler, *MT* Maintenance-treated at screening, *NMT* Not maintenance-treated at screening, *SD* Standard deviation, *SE* Standard error, *SGRQ* St George’s Respiratory Questionnaire

GFF MDI reduced total SGRQ score from baseline by 4.397 and 4.126 points for NMT and MT patients at Week 24, respectively (Table 2). In the overall ITT population, more patients treated with GFF (40.8%) achieved a minimal clinically important difference (MCID, defined as ≥4.0 units of change from baseline) in SGRQ at Week 24 compared with FF (38.1%), GP (35.0%), and placebo (30.9%). Rescue salbutamol use was reduced by 1.5 puffs/day over 24 weeks compared to baseline in subjects treated with GFF in both NMT and MT cohorts (Table 2), and was significantly reduced compared to placebo (*P* < 0.0001).

**Fig. 3:**
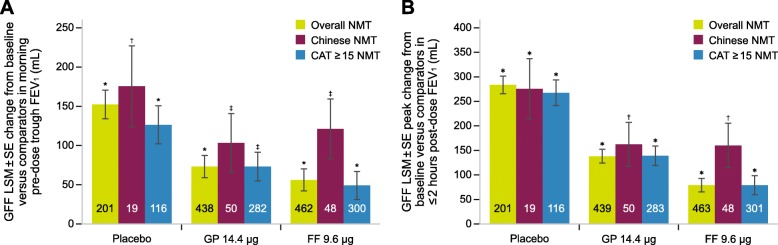
Trough FEV_1_ (**a**) and peak post-dose FEV_1_ (**b**) in Chinese and symptomatic NMT patients. Data labels within columns are N number. * *P* < 0.0001, ^†^*P* ≤ 0.001, and ^‡^*P* ≤ 0.01, compared with GFF MDI. All P-values < 0.05 should only be interpreted in terms of nominal significance as the analyses in these subgroups were exploratory and post-hoc. CAT = COPD Assessment Test; COPD = chronic obstructive pulmonary disease; FEV_1_ = forced expiratory volume in 1 s; FF = formoterol fumarate; GFF = glycopyrrolate/formoterol fumarate; GP = glycopyrrolate; LSM = least squares mean; MDI = metered dose inhaler; NMT = not maintenance-treated at screening; SE = standard error

Symptomatic NMT patients treated with GFF MDI demonstrated improved lung function at Week 24, consistent with the overall NMT population. Change from baseline in trough FEV_1_ was 126 mL with GFF MDI versus placebo (*P* < 0.0001), 49 mL versus FF (*P* = 0.0059), and 73 mL versus GP (*P* < 0.0001). For Chinese NMT patients, corresponding values for changes from baseline trough FEV_1_ were 175 mL (*P* = 0.0008), 121 mL (*P* = 0.0017), and 103 mL (*P* = 0.0065), respectively (Fig. 3a). GFF MDI also improved peak change from baseline in FEV_1_ in these subgroups; symptomatic NMT: 267 mL (*P* < 0.0001) versus placebo, 79 mL (*P* < 0.0001) versus FF, and 139 mL (*P* < 0.0001) versus GP; Chinese NMT: 275 mL (*P* < 0.0001) versus placebo, 160 mL (*P* = 0.0005) versus FF, and 162 mL (*P* = 0.0004) versus GP (Fig. 3b).

No new safety signals were identified from the pooled analysis. In total, 55.5 and 57.2% of patients experienced ≥1 treatment-emergent adverse event (TEAE) in the NMT and MT cohorts, respectively, with the majority considered non-serious and unrelated to study treatment (Table 3). Treatment discontinuation following TEAEs were limited, ranging from 4.6% (FF) to 5.5% (GFF) of NMT subjects, and 5.7% (FF) to 7.1% (placebo) of MT subjects. Three deaths occurred in the NMT cohort (one each in placebo, FF, and GFF subjects), and seven occurred in the MT cohort (one each in placebo, GP, and FF subjects, and four in GFF subjects) over the study period; none were considered treatment-related.

**Table 3:** Summary of adverse events

	NMT				MT			
Treatment	GFF (***n*** = 638)	FF (***n*** = 548)	GP (***n*** = 516)	Placebo (***n*** = 241)	GFF (***n*** = 947)	FF (***n*** = 812)	GP (***n*** = 846)	Placebo (***n*** = 435)
**TEAEs, n (%)**								
Patients with ≥1 TEAE	362 (56.7)	304 (55.5)	280 (54.3)	133 (55.2)	560 (59.1)	457 (56.3)	469 (55.4)	252 (57.9)
Patients with serious TEAEs	43 (6.7)	33 (6.0)	35 (6.8)	18 (7.5)	90 (9.5)	73 (9.0)	72 (8.5)	32 (7.4)
Deaths (all-cause) during treatment period	1 (0.2)	1 (0.2)	0 (0)	1 (0.4)	4 (0.4)	1 (0.1)	1 (0.1)	1 (0.2)
**TRAEs, n (%)**								
Patients with TEAEs related to study treatment	75 (11.8)	58 (10.6)	51 (9.9)	19 (7.9)	97 (10.2)	86 (10.6)	99 (11.7)	50 (11.5)
Patients with serious TEAEs related to study treatment	7 (1.1)	1 (0.2)	6 (1.2)	1 (0.4)	3 (0.3)	7 (0.9)	9 (1.1)	2 (0.5)
**Common TEAEs, n (%)**								
Upper respiratory tract infection	28 (4.4)	20 (3.6)	26 (5.0)	13 (5.4)	42 (4.4)	39 (4.8)	41 (4.8)	29 (6.7)
Viral upper respiratory tract infection	31 (4.9)	28 (5.1)	20 (3.9)	10 (4.1)	44 (4.6)	43 (5.3)	41 (4.8)	16 (3.7)
Dyspnea	11 (1.7)	8 (1.5)	7 (1.4)	7 (2.9)	24 (2.5)	27 (3.3)	25 (3.0)	19 (4.4)
Nasopharyngitis	14 (2.2)	14 (2.6)	9 (1.7)	10 (4.1)	31 (3.3)	15 (1.8)	16 (1.9)	9 (2.1)
Back pain	17 (2.7)	12 (2.2)	10 (1.9)	9 (3.7)	19 (2.0)	13 (1.6)	19 (2.2)	2 (0.5)
Cough	23 (3.6)	11 (2.0)	14 (2.7)	6 (2.5)	31 (3.3)	21 (2.6)	23 (2.7)	8 (1.8)
COPD	14 (2.2)	11 (2.0)	16 (3.1)	5 (2.1)	26 (2.7)	19 (2.3)	26 (3.1)	15 (3.4)
Bronchitis	9 (1.4)	5 (0.9)	9 (1.7)	2 (0.8)	15 (1.6)	13 (1.6)	26 (3.1)	15 (3.4)
Hypertension	14 (2.2)	5 (0.9)	6 (1.2)	8 (3.3)	14 (1.5)	16 (2.0)	14 (1.7)	16 (3.7)
Headache	15 (2.4)	17 (3.1)	11 (2.1)	3 (1.2)	15 (1.6)	18 (2.2)	20 (2.4)	4 (0.9)

Common TEAEs defined as those occurring in ≥ 3% of patients in any treatment arm. *COPD* Chronic obstructive pulmonary disease, *FF* Formoterol fumarate, *GFF* Glycopyrrolate and formoterol fumarate, *GP* Glycopyrrolate, *MT* Maintenance-treated at screening, *NMT* Not maintenance-treated at screening, *TEAE* Treatment-emergent adverse event, *TRAE* Treatment-related adverse event

## Discussion

This *post-hoc* analysis is the first to compare GFF MDI with its monocomponents in patients grouped by prior exposure to maintenance therapy. Long-acting dual bronchodilators are primarily recommended for step-up treatment following unsatisfactory disease control with first-line LABA or LAMA monotherapy. However, many patients indicated for monotherapy experience ongoing symptoms and respiratory decline during treatment [1, 5, 6]. Analyses examining whether patients may benefit from commencing LAMA/LABA therapy directly are emerging, but none have included GFF [15–20]. In our analysis, GFF MDI significantly improved respiratory outcomes for both NMT and MT cohorts compared with monocomponents and placebo, without increasing safety concerns. This suggests that GFF MDI may be used as first-line therapy, achieving similar safety and efficacy to current use. Improved stratification is needed to identify patients who may benefit from direct dual therapy, to avoid over-medication in those who could be managed sufficiently with a single LAMA or LABA.

In our analysis, GFF MDI provided greater improvement to morning pre-dose trough and post-dose peak FEV_1_ measures from baseline, compared with monocomponents and placebo in both NMT and MT subjects at Week 24 and over 24 weeks. GFF exceeded the MCID for morning pre-dose trough FEV_1_ in both cohorts [21]. These findings are consistent with previous primary analyses of the PINNACLE studies, and consistent for the symptomatic and Chinese NMT subgroups.

Currently, first-line LAMA/LABA combinations are only recommended for highly symptomatic subsets of GOLD B and D patients [1]. Unlike trials in some other LAMA/LABA fixed-dose combinations [17, 22], the PINNACLE studies were not restricted to symptomatic patients. A recent pooled analysis found that respiratory benefits for the total population were upheld in GOLD A PINNACLE patients, classified as those with CAT < 10/modified Medical Research Council scale of 0–1, ≤1 moderate exacerbation in the previous year, and no exacerbations leading to hospitalization in the previous year, suggesting GFF MDI is suitable for patients with milder symptoms and low exacerbation risk [23]. In our analysis, 14% of patients in the NMT and MT cohorts were classified as GOLD A. Hence, our findings support the safety and efficacy of first-line GFF MDI for patients with varying symptom burden.

CID is a composite measure of functional and patient-reported outcomes, indicative of worsening COPD and linked to poor long-term outcomes [19]. GFF reduced the risk of CID by 17 and 21% compared with FF and GP, respectively, and by 43% compared with placebo in NMT subjects, suggesting the combination more effectively stabilizes outcomes representative of disease progression. This trend was upheld in MT patients. SGRQ total score, which feeds into the CID outcome as a quality of life measure, was also improved by GFF MDI for both cohorts. CID improvements in NMT patients were not driven by exacerbation differences, as this group included predominately GOLD B patients who are at low exacerbation risk.

In our study, rescue salbutamol use was reduced by 1.5 puffs/day (approximately one-third of baseline puffs/day) over 24 weeks in NMT and MT patients treated with GFF MDI. All comparators reduced salbutamol use compared with placebo. A recent systematic review of 46 studies found that changes in rescue medication use are associated with important COPD measures, including SGRQ score, dyspnea, exacerbation rate, and trough FEV_1_ [24].

No further safety findings emerged from the analysis. TEAEs and TEAE-related discontinuations were similar across all treatment groups and populations. These findings concur with other meta-analyses and systematic reviews, which continue to find no additional or synergistic safety signals when LAMAs and LABAs are used in combination [25, 26].

Preliminary analyses of other LAMA/LABA combinations demonstrate that first-line dual therapy may be more efficacious than monotherapy. Maximizing bronchodilation is a key outcome of maintenance therapy. Previous analyses of monotherapies in various maintenance treatment-naïve populations have found similar safety and efficacy results to unsorted populations [2–4]. To our knowledge, maintenance treatment-naïve populations have achieved greater respiratory improvements with dual bronchodilators over monotherapy in all studies to date. A pre-specified subgroup analysis of maintenance treatment-naïve and maintenance-treated patients treated with umeclidinium/vilanterol reported significant improvements in trough FEV_1_, reaching MCID in NMT patients [15]. Better respiratory improvements with dual bronchodilation were found for maintenance treatment-naïve patients in retrospective studies comparing umeclidinium/vilanterol to tiotropium, and tiotropium/olodaterol to tiotropium and placebo [16, 17]. Long-acting bronchodilator-naïve patients treated with indacaterol, glycopyrronium, or a combination also achieved optimal respiratory outcomes via a combination approach [18]. First-line dual bronchodilation has been shown to reduce exacerbation risk, dyspnea, risk of CID, and rescue medication use, whilst improving patient-reported symptom scores, health-related quality of life, and SGRQ scores [15–20, 27]. These data lend further support for the preferential use of combination treatments for first-line COPD maintenance therapy.

All but one [15] study in populations with lapsed or no prior history of maintenance treatment, including our own, have been designed *post-hoc.* These findings are hence provisional, and there remains a paucity of prospective data. Nonetheless, coupled with the understanding that effective maintenance therapy should be commenced promptly to prevent the increased rate of pulmonary decline observed in early disease [3, 28–33], these findings support the case for commencing COPD patients directly on LAMA/LABA to improve symptoms and preserve lung function.

Limitations of our study include that the PINNACLE trials were not prospectively powered with NMT and MT subgroups in mind. Prospective studies of first-line dual bronchodilator therapy are required. More targeted subgroups would also be of interest; future research should examine outcomes in patients who only briefly receive LABA or LAMA monotherapy prior to escalation, a common clinical occurrence, compared to those directly prescribed dual therapy. Similarly, future research should assess first-line dual LAMA/LABA treatment in real-world populations.

## Conclusions

Our analysis found important efficacy advantages, with no safety, tolerability, or efficacy disadvantages to commencing GFF MDI in the NMT population. These data add to the growing body of evidence supporting LABA/LAMA combination therapies for the first-line treatment of moderate-to-very severe COPD in appropriate patients. Validation of these findings with prospective and real-world studies are required.

## Data Availability

Data underlying the findings described in this manuscript may be obtained in accordance with AstraZeneca’s data sharing policy described at: https://astrazenecagrouptrials.pharmacm.com/ST/Submission/Disclosure.

## Abbreviations

CAT: COPD Assessment Test
CI: Confidence interval
CID: Clinically important deterioration
COPD: Chronic obstructive pulmonary disease
FEV_1_: Forced expiratory volume over 1 s
FF: Formoterol fumarate
GFF: Glycopyrrolate/formoterol fumarate
GOLD: Global Initiative for Chronic Obstructive Lung Disease
GP: Glycopyrrolate
ICS: Inhaled corticosteroid
ITT: Intent to treat
LABA: Long-acting beta-agonist
LAMA: Long-acting muscarinic antagonist
MCID: Minimal clinically important difference
MDI: Metered dose inhaler
MT: Maintenance-treated at screening
NMT: Not maintenance-treated at screening
SGRQ: St George’s Respiratory Questionnaire
TEAE: Treatment-emergent adverse event
